# *Pinus sylvestris* bark extract reduces the impact of *Heligmosomoides bakeri* infection on C57BL/6 but not on BALB/c mice (*Mus musculus*)

**DOI:** 10.1017/S0031182024001148

**Published:** 2024-11

**Authors:** Berit Marie Blomstrand, Stig Milan Thamsborg, Håvard Steinshamn, Heidi Larsen Enemark, Inga Marie Aasen, Karl-Christian Mahnert, Kristin Marie Sørheim, Francesca Shepherd, Jos Houdijk, Spiridoula Athanasiadou

**Affiliations:** 1 Norwegian Centre for Organic Agriculture, Tingvoll, Norway; 2 Department of Veterinary and Animal Sciences, University of Copenhagen, Frederiksberg, Denmark; 3 Division of Food Production and Society, Grassland and Livestock, Norwegian Institute of Bioeconomy Research, Tingvoll, Norway; 4 Department of Animal Health and Food Safety, Norwegian Veterinary Institute, Oslo, Norway; 5 Department of Animal and Veterinary Sciences, Aarhus University, Tjele, Denmark; 6 SINTEF Industry, Biotechnology and Nanomedicine, Trondheim, Norway; 7 The Norwegian Institute of Wood Technology, Oslo, Norway; 8 Department of Animal and Veterinary Sciences, Scotland's Rural College (SRUC), Edinburgh, UK

**Keywords:** bark, condensed tannins, gastrointestinal nematodes, plant secondary metabolites, resistance, tolerance

## Abstract

Plant secondary metabolites (PSMs) may improve gastrointestinal health by exerting immunomodulatory, anti-inflammatory and/or antiparasitic effects. Bark extracts from coniferous tree species have previously been shown to reduce the burden of a range of parasite species in the gastrointestinal tract, with condensed tannins as the potential active compounds. In the present study, the impact of an acetone extract of pine bark (*Pinus sylvestris*) on the resistance, performance and tolerance of genetically diverse mice (*Mus musculus*) was assessed. Mice able to clear an infection quickly (fast responders, BALB/c) or slowly (slow responders, C57BL/6) were infected orally with 200 infective third-stage larvae (L_3_) of the parasitic nematode *Heligmosomoides bakeri* or remained uninfected (dosed with water only). Each infection group of mice was gavaged for 3 consecutive days from day 19 post-infection with either bark extract or dimethyl sulphoxide (5%) as vehicle control. Oral administration of pine bark extract did not have an impact on any of the measured parasitological parameter. It did, however, have a positive impact on the performance of infected, slow-responder mice, through an increase in body weight (BW) and carcase weight and reduced feed intake by BW ratio. Importantly, bark extract administration had a negative impact on the fast responders, by reducing their ability to mediate the impact of parasitism through reducing their performance and tolerance. The results indicate that the impact of PSMs on parasitized hosts is affected by host's genetic susceptibility, with susceptible hosts benefiting more from bark extract administration compared to resistant ones.

## Graphical abstract

**Figure:**
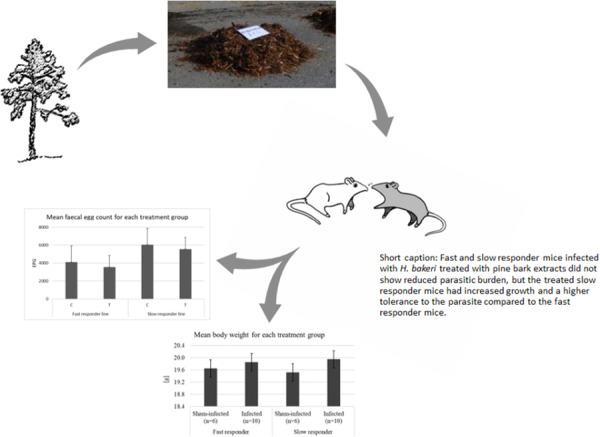

## Introduction

Infections caused by gastrointestinal nematodes (GINs) have a negative impact on animal health and welfare, productivity and overall farm profitability worldwide (Charlier *et al.*, 2020). Anthelmintic pharmaceuticals are crucial for the control of GINs and often represent the most easily available option (Molento, 2009). However, extensive use of anthelmintic pharmaceuticals has led to widespread anthelmintic resistance in GINs (Vineer *et al.*, 2020). This worldwide challenge has been known for decades; thus there is a pressing need for alternative options for the control of GINs. Among the alternatives currently under investigation, plant secondary metabolites (PSMs) have been extensively researched *in vitro* and *in vivo* for their antiparasitic properties (Anthony *et al.*, 2005; Hoste *et al.*, 2015; Spiegler *et al.*, 2017). Some studies have shown that polyphenols such as condensed tannins (CT) have anthelmintic activity (Hoste *et al.*, 2006; Desrues *et al.*, 2016; Mengistu *et al.*, 2017), whereas other studies have revealed other plant compounds such as sesquiterpene lactones, which may be responsible for antiparasitic attributes of plant extracts (Valente *et al.*, 2021).

Bark extracts from coniferous trees are rich in PSMs such as CT, and our recent investigations have shown that bark extracts have antiparasitic properties *in vitro* (Athanasiadou *et al.*, 2021; Blomstrand *et al.*, 2021). We have shown that CT in conifer bark were at least partly responsible for the antiparasitic efficacy against *Trichostrongylus colubriformis* but other compounds may also have contributed to activity (Chylinski *et al.*, 2023).

In this study, the anthelmintic activity of bark extract was tested for the first time *in vivo*, in 2 different lines of mice infected with the intestinal nematode *Heligmosomoides bakeri*. *Heligmosomoides bakeri* is a trichostrongyloid nematode of the house mouse, *Mus musculus*, placed phylogenetically in the same order as some of the most pathogenic nematode species in humans and livestock (*Ancylostoma duodenale*, *Necator americanus*, *Ostertagia* spp., *Haemonchus contortus*, *Trichostrongylus* spp., etc.) (Gouÿ De Bellocq *et al.*, 2001; Reynolds *et al.*, 2012). It has a direct life cycle with a pre-patent time of 9–11 days and is widely used as a laboratory model, to investigate mechanisms of host resistance and performance to GINs infections (Monroy and Enriquez, 1992; Behnke *et al.*, 2009). In the present study, this model was used to quantify the impact of bark extract administration on the resistance, performance and tolerance of genetically diverse mice.

Host resistance, i.e. the ability of hosts to limit the parasite burden, is affected by many factors, including host nutrition (Houdijk *et al.*, 2012), but is largely under genetic control (Råberg *et al.*, 2008; Råberg, 2014). Behnke *et al.* (2006) demonstrated that there is genetic variation in the susceptibility of specific mouse lines towards *H. bakeri*. Mice strains such as BALB/c are considered fast responders, as they quickly clear out *H. bakeri* infection, as measured by fecal egg counts (FEC) and pathogen load compared to others, such as C57BL/6, which are slow responders. The latter are maintaining the parasite population in their intestine for many weeks prior to expulsion. Host tolerance is the ability of the host to minimize the detrimental impact of infection on performance and is measured as the regression of performance on pathogen burden (Mulder and Rashidi, 2017). Tolerance is less investigated compared to resistance, but it also appears to be under genetic control. Athanasiadou *et al.* (2015) showed that genetically diverse lines of mice demonstrated differences in their tolerance to *H. bakeri* too, with BALB/c mice suffering less from the consequences of parasitism on their performance compared to C57BL/6 mice. Resilience is the host's ability to maintain performance (e.g. as measured by body weight (BW)) when exposed to pathogens, but unlike tolerance, it does not need records of pathogen load (Mulder and Rashidi, 2017).

The hypothesis here was that infected, slow-responder mice (C57BL/6) would benefit more from the bark extract administration compared to fast-responder mice (BALB/c). The prediction was that infected, bark extract-treated, slow-responder mice would experience improved resistance, as measured by a reduction in the parasitic burden and FEC, better performance and improved tolerance to the parasitic infection compared to the fast-responder mice.

## Materials and methods

### Experimental animals and housing

Female, 5 weeks old BALB/c (*n* = 48) and C57BL/6 (*n* = 48) mice (obtained from Envigo and Charles Rivers, respectively) were housed in pairs in standard transparent, solid bottom Home Office approved cages under standard environmental conditions (21 ± 1°C, relative humidity 45 ± 5%, 12 h light–dark cycle) with fresh sawdust bedding provided weekly. A Plexiglass cylinder and shredded paper were provided as environmental enrichment. All animals were offered a maintenance diet (14% crude protein, Special Diet Services, Lillico Biotechnologies, UK) and water *ad libitum* throughout the experiment.

### Infective larvae (L_3_) of *H. bakeri*

*Heligmosomoides bakeri*-infective L_3_ were cultured from mono-specifically infected donor mice and harvested 7 months prior to infection. They were stored at 2–5°C in distilled water (dH_2_O) until use. One week before infecting the mice, the larvae were washed, re-baermanized, counted and the concentration was set at 1000 L_3_ mL^−1^ dH_2_O.

### Bark extraction and CT determination

Bark from *Pinus sylvestris* (Scots pine), a coniferous tree species of the family *Pinaceae*, was ring debarked and collected in a sawmill in eastern Norway (Bergene Holm AS, Kirkenær) in March 2017 and stored at −20°C until processing. The bark was milled to chips of 5–20 mm in a hammer mill (Schutte Mini Mill, Buffalo, NY, USA) and freeze-dried. The extract was prepared by adding 1200 mL aqueous acetone (70%) to 150 g ground bark, followed by incubation for 1 h in a water bath (40°C) with slow stirring. The extract was filtered through a filter cloth and the bark was extracted for a second time with 1250 mL acetone (70%) for 30 min. Finally, both extract volumes were combined, and acetone was removed by evaporation (rotavapor, 40°C) before freeze-drying. Total CT was quantified by the butanol-HCl assay. The freeze-dried extract was dissolved in methanol (80% in water) and analysed with cyanidin-HCl as standard, using the conventional reagent without acetone, 2.5 h, and reading the absorbance at 545 nm (Grabber *et al.*, 2013).

To reconstitute the freeze-dried extract prior to administration to mice, dimethyl sulphoxide (DMSO) was used. As pure DMSO is toxic both to mice and the parasite, the dry bark extract was dissolved in 5% DMSO in dH_2_O and shaken on a shaker for 48 h (21°C) to achieve 150 mg bark extract dry matter (DM) mL^−1^ in 5% DMSO (Worthley and Schott, 1969). This reconstituted extract was used as the high bark dose, as shown in the experimental design (Table 1), and a 1:1 dilution of this was used for the low bark extract dose. Following reconstitution, the same method as previously described (Grabber *et al.*, 2013) was used to determine the final CT concentration received by the animals.

**Table 1.** Experimental setup of the *in vivo* experiment

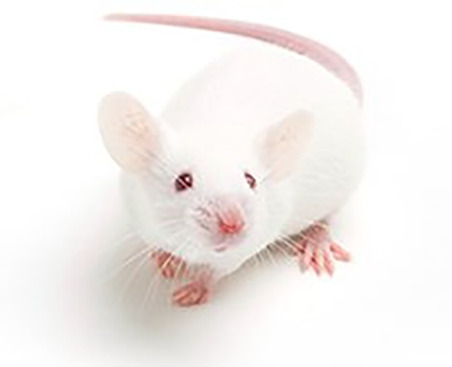						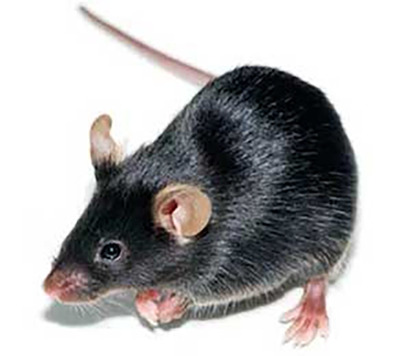					
Fast-responder line, *n* = 48						Slow-responder line, *n* = 48					
Sham-infected, *n* = 18			Infected, *n* = 30			Sham-infected, *n* = 18			Infected, *n* = 30		
0 mg mL^−1^ *n* = 6	75 mg mL^−1^ *n* = 6	150 mg mL^−1^ *n* = 6	0 mg mL^−1^ *n* = 10	75 mg mL^−1^ *n* = 10	150 mg mL^−1^ *n* = 10	0 mg mL^−1^ *n* = 6	75 mg mL^−1^ *n* = 6	150 mg mL^−1^ *n* = 6	0 mg mL^−1^ *n* = 10	75 mg mL^−1^ *n* = 10	150 mg mL^−1^ *n* = 10

Fast- (BALB/c) and slow-responder (C57BL/6) to a *Heligmosomoides bakeri* infection mice were infected with 200 L_3_ (or remained as uninfected controls) and treated orally with 200 μL of acetone extracted pine bark extract dissolved in 5% DMSO (or 200 μL of 5% DMSO). The mice were treated with bark extract concentrations at 0, 75 and 150 mg DM mL^−1^.

DMSO, dimethyl sulphoxide; DM, dry matter bark extract.

### Experimental design

Within each genetic line, animals were randomly allocated in treatment groups based on their arrival BW, with 10 animals allocated in each infected group and 6 animals in each non-infected control group (Table 1). Power calculation indicated that to detect a difference in worm counts between the groups of animals administered the bark extracts or control of the magnitude of 40% at a level of significance of *P* = 0.05, sample size should be set at 10. The animals were acclimatized in their respective cages for 1 week prior to the *H. bakeri* infection (day 0).

The experiment was executed in 2 blocks, with half the animals in each block (balanced across group treatments). The second block started 3 days after the first block, to facilitate post-mortem examinations. For feed intake (FI) measurements, the experimental unit was the cage, i.e. the 2 animals in each cage. For all other measurements, the experimental unit was the individual animal. On day 0, mice were infected with 200 *H. bakeri* L_3_ (in 200 μL of water) by oral gavage; a bulb-tipped gastric gavage needle and 1 mL syringe were used for this purpose. The non-infected mice were given 200 μL of water.

All mice received 200 μL of the bark extract in 5% DMSO (or 5% DMSO only for the negative control animals) for 3 consecutive days, days 19–21 post-infection. Three levels of bark were tested: no bark extract (5% DMSO), low (75 mg mL^−1^ in 5% DMSO) or high (150 mg mL^−1^ in 5% DMSO) concentration of the bark extract, equivalent to 0.75 g DM kg^−1^ BW and 1.5 g DM kg^−1^ BW, respectively. The selection of the levels of supplementation was based on studies where *H. bakeri*-infected mice were treated with other plant extracts or from results extrapolated from studies where bark extracts were tested on other host species (Morais-Costa *et al.*, 2016; Tolossa *et al.*, 2021; Blomstrand *et al.*, 2022).

### Measurements and sample collection

#### Parasitological measurements

Parasitological measurements such as FEC (eggs per gram (EPG)), eggs in colon (EIC) and total worm counts (TWC) were collected as indicators of resistance (Råberg *et al.*, 2008). Individual fecal samples were collected at 4 time points throughout the study from day 14 onwards (days 14, 17, 22 and 28). On each of these occasions mice were individually placed in a clean cage for a minimum of 20 min and feces were collected, weighed and processed for FEC determination within 48 h, using a flotation method (Christie and Jackson, 1982). On day 28, mice were sedated through increasing CO_2_ inhalation and euthanized by CO_2_ asphyxiation. The colon contents were removed, weighed and analysed for FEC, and this figure was multiplied by the colon content to obtain EIC. To recover the adult *H. bakeri*, the small intestine (SI) was opened longitudinally and placed in phosphate-buffered saline at 37°C for 3 h, to allow the worms to migrate out of the tissue. SI and recovered worms were then fixed in 70% ethanol for sex determination and counting. *Per capita* fecundity was calculated by dividing EIC by the total number of female parasites recovered.

#### Performance measurements and tolerance calculation

BW, feed offered and feed refused were measured regularly throughout the experiment, every 3–5 days. To calculate the daily FI per cage, feed refusals and fresh feed in were weighed. BW, carcase weight (CW) and FI were used as indicators of performance (growth) and were also used for tolerance calculations. In a previous study, CW has been shown to be a more reliable indicator of performance and tolerance than BW, as it disregards the increase in the weight of internal organs in parasitized mice attributed to inflammation (Athanasiadou *et al.*, 2015). The FI–BW ratio provides information about the efficiency of feed utilization and was calculated for the pre-infection period and days 3, 11, 15, 19, 23 and 28 in the post-infection period. At post-mortem, the weight of the spleen (as an organ primarily responsible for the immune response), SI (as the parasite niche and a site for local inflammation) and CW were recorded. To quantify the effect of bark extract administration on spleen and SI weight, spleen–CW and SI–CW ratios were calculated. This was performed to take into consideration the differences in the size of the animals; analysis showed that the impact of bark extract treatment was not affected by animal size (results not shown), and it was refrained from using the ratios in the final analysis. Additional Pearson's correlations were performed to associate TWC and spleen weight in mice, as an extra measurement of resistance. Previous evidence has shown that spleen mass and parasite load are negatively associated in resistant deer (Corbin *et al.*, 2007).

Tolerance estimates were calculated from Pearson's correlations on transformed, normalized CW, TWC, FEC (day 28) and EIC data for each treatment group (Athanasiadou *et al.*, 2015). A significant negative correlation between a parasitological indicator and CW (e.g. high parasite load associated with reduction in CW) would indicate low host tolerance, i.e. low ability to withstand the penalties of parasitism. If this relationship was non-significant (e.g. high parasite load associated with no change in CW) or positive, it would be indicative of high host tolerance, i.e. the host does not suffer from the penalties of parasitism.

### Statistical analyses

For all analysis, the experimental block was included in the model as a factor. For FI and FI–BW ratio, the experimental unit was the cage. For all other data, the experimental unit was the animal. Data were analysed by using the MIXED procedure in SAS (SAS 9.3, 2014, SAS Institute Inc., Cary, NC, USA). As there was no significant difference between the groups administered the 2 levels of bark extracts, all data from the bark extract-treated groups were pooled. For the analysis of the variables FEC, BW, FI and FI–BW ratio, the study was split into 2 periods: the pre-treatment period (days 0–18 post-infection) and the post-treatment period (days 19–28 post-infection). The effects of line (BALB/c or C57BL/6), infection (infected or sham-infected with *H. bakeri*), bark administration (with or without bark extract) and day in the post-treatment period, and their interactions were treated as fixed effects, and animal within the cage as a random effect. The average values of BW and FI in the pre-infection period (days −7 to 0) were included as covariates for BW, CW and SI, and for spleen weight, and FI, respectively, and variation within animal was accounted for an analysis of repeated measures. For data obtained at post-mortem, the effects of line (BALB/c or C57BL/6), infection (infected or sham-infected with *H. bakeri*) and bark administration (with or without bark extract) and their interactions were used as fixed effects and animal within cage as random effect. The optimal covariance structure was assessed for each dependent variable with attention to Akaike's information criterion. Differences between least square means of response variables were estimated with Tukey's test. Results were considered significant at *P* < 0.05.

If the residual variance of the data was not constant, the variables were transformed, either *via* Box–Cox transformation (FEC: lambda = 0.2, EIC: lambda = 0.25), or logarithmically (log10; weight of SI and spleen, TWC and female worm fecundity). Tests of significance were performed on the transformed scale and then back-transformed to the original scale for presentation. Predicted means on the transformed scale, when back-transformed, provide predicted medians on the original scale. Because standard errors are not constant for comparison on the original scale, the results for the transformed variables are presented as least squares means with pooled standard errors based on the original values.

To test the impact of bark extract on tolerance of mice, the data were transformed and tested in R (v. 4.1.0) and RStudio (v. 1.4.1717).

## Results

### CT concentration in the bark extracts

The CT concentration in the acetone pine bark extract was 80.3 mg g^−1^ DM extract (±4.2). Based on this measurement, the expected concentration of CT for the high and low bark extracts was 12 and 6 mg CT mL^−1^, respectively. The analyses of the reconstituted extracts (in 5% DMSO) however, showed that the mean CT concentration administered to each animal was 4.2 and 2.0 mg CT mL^−1^ (s.e.m. 0.450 and 0.038) for the high and low bark doses, respectively. As each animal received 0.2 mL of reconstituted extract, this equalled approximately 0.84 and 0.40 mg CT day^−1^ (42 and 20 mg CT kg^−1^ BW), when receiving the high or low bark extract doses, respectively.

### Bark extract administration did not have an impact on parasite load and fecundity in mice

Nematode eggs were first observed on day 14 and peaked on day 17 in both mouse lines. The administration of bark extracts had no effect on the measured parasitological parameters (*P* > 0.1, Table 2). There was a significant effect of the line on FEC, EIC and TWC during the post-treatment period; the slow-responder line had a greater FEC, EIC and TWC compared to the fast-responder line (*P* < 0.05; Table 2). There was a significant line by day interaction in FEC, where the rate of reduction in the fast-responder line was greater compared to the rate of reduction in the slow-responder line (*P* < 0.05), with 2159 EPG and 757 EPG for days 22 and 28, respectively, for the fast-responder line and 2370 EPG and 2016 EPG for days 22 and 28, respectively, for the slow-responder line. There was a tendency of greater female worm fecundity in the slow-responder line compared to the fast-responder line (*P* = 0.07).

**Table 2.** Effect of bark extract administration (T; days 19–21 post-infection) during the post-treatment period (PT), on FEC, TWC, EIC and eggs per female nematode (fecundity) in fast- and slow-responder mice infected with *H. bakeri* larvae

Parameter^a^ (mean)	Fast-responder line		Slow-responder line		s.e.m.		*P* value				
	C	T	C	T	C	T	Line	Extract	Line × extract	Day	Line × day
FEC	4095	3548	6035	5544	1821	1304	0.0453	0.57	0.90	<0.0001	0.0044
TWC	14	14	33	41	17.3	12.2	0.0155	0.82	0.76	n.a.	n.a.
EIC	332	329	865	1521	598	423	0.0041	0.42	0.41	n.a.	n.a.
Fecundity	28.6	28.0	39.2	42.2	8.2	6.1	0.0698	0.89	0.80	n.a.	n.a.

FEC, mean fecal egg count during the post-treatment period; TWC, mean total worm count in the small intestines; EIC, mean eggs in total colon content; fecundity, EIC per female nematode; C, negative controls (infected, untreated; *n* = 10); T, infected, bark extract-treated groups, *n* = 20; extract: extract treatment; n.a., not applicable.

a All data except for FEC were obtained at necropsy, on day 28. FEC was obtained on samples collected days 22 and 28. All data were back-transformed, and the standard error of the mean (s.e.m.) was calculated on the original data.

For both fast- and slow-responder lines, a positive correlation between TWC and spleen weight was identified (*r* = 0.58 and 0.46, respectively, *P* < 0.05). There was a significant positive correlation between TWC and the spleen weight for the treated, fast-responder line and for the untreated, slow-responder line. There was no such correlation for the untreated, fast-responder line, but for the treated, slow-responder line there was a positive correlation that tended to be significant (*r* = 0.4, *P* = 0.08).

### Bark extract administration improved the performance of infected, slow-responder mice but reduced the performance of fast-responder mice

Animals in all experimental groups continued to grow throughout the whole experimental period. The average growth was greater for the pre-infection period (0.45 g day^−1^) compared to the post-infection period across all groups (0.11 g day^−1^). During the pre-treatment period, infection had no impact on BW, FI or FI–BW ratio on any of the mouse lines (*P* > 0.1). The mean BW of the fast-responder mice during this period was 18.3 g, and that of the slow-responder mice was 18.5. FI during P1 was greater (*P* < 0.05) for the fast-responder (3.25 g day^−1^) compared to the slow-responder (3.07 g day^−1^) mice.

**Figure 1.**
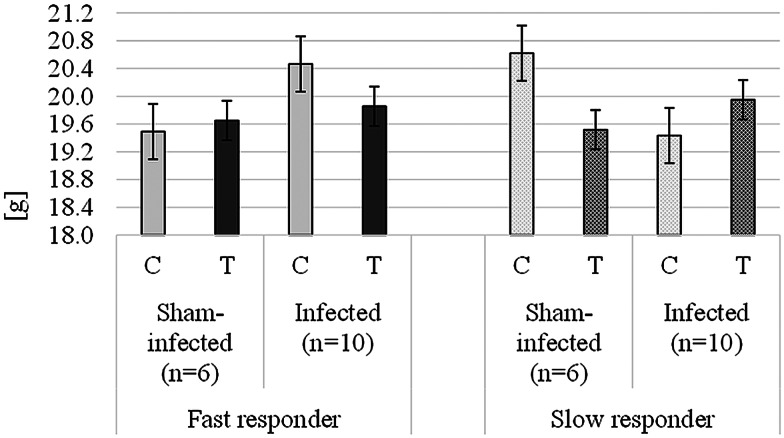
Mean body weight (BW; back-transformed) for the post-treatment period (days 19–28) for sham-infected and infected, fast- and slow-responder mouse lines not treated and treated with bark extracts. C, untreated groups (light grey); T, treated groups (dark grey); pattern fill, slow-responder mice; solid fill, fast-responder mice; error bars, standard error of the mean.

Performance measurements during the post-treatment period are presented in Table 3. During this period, a significant 3-way line × infection × extract interaction was evident on the mean BW and CW (*P* < 0.05). Bark extract administration had a negative impact on the BW of infected, fast-responder mice whereas it had a positive impact on the BW and CW of infected mice in the slow-responder line, compared to their respective untreated controls (Fig. 1). Furthermore, the administration of bark extract did not have any impact on the BW and CW of the sham-infected, fast-responder mice whereas it had a negative impact on the sham-infected, slow-responder mice. There was no effect of line on BW (*P* > 0.1); infection alone had no impact on BW and CW during the post-treatment period (*P* > 0.1).

**Table 3.** Effect of bark extract administration (T; days 19–21 post-infection) during the post-treatment period (PT) on feed intake (FI), body weight (BW), carcase weight (CW), spleen weight and small intestine (SI) weight in fast- and slow-responder mice sham infected or infected with *H. bakeri* larvae

Parameters	Unit	Fast-responder mice				Slow-responder mice				s.e.m.		*P* value									
		Sham		Inf.		Sham		Inf.		C	T	Cov.	Line	Inf.	Line × Inf.	Ext.	Line × Ext.	Inf. × Ext.	Line × Inf. × Ext.	Day	Day × line
		C	T	C	T	C	T	C	T												
FI–PT	g	3.4	3.2	3.4	3.4	3.1	3.0	2.9	2.9	0.10	0.07	0.701	<0.001	0.484	0.027	0.345	0.466	0.377	0.660	0.137	0.996
BW–PT	g	19.5	19.6	20.5	19.9	20.6	19.5	19.4	20.0	0.40	0.28	0.004	0.952	0.666	0.057	0.288	0.890	0.387	0.019	<0.001	0.035
FI:BW–PT	g g^−1^	0.17	0.16	0.16	0.17	0.15	0.16	0.15	0.15	0.004	0.003	0.135	<0.001	0.576	0.540	0.723	0.414	0.817	<0.001	0.361	0.665
CW	g	14.2	14.3	14.4	14.0	15.4	14.8	14.5	14.7	0.24	0.17	<0.001	<0.001	0.046	0.134	0.274	0.942	0.496	0.030	n.a.	n.a.
Spleen	g	0.09	0.10	0.15	0.15	0.07	0.07	0.08	0.09	0.035	0.025	0.481	<0.001	<0.001	0.021	0.634	0.857	0.206	0.226	n.a.	n.a.
SI	g	1.48	1.60	2.26	2.10	1.30	1.22	1.62	1.62	0.022	0.015	0.889	<0.001	<0.001	0.114	0.644	0.599	0.477	0.077	n.a.	n.a.

Sham, non-infected group (*n* = 18); Inf., infected group (*n* = 30); C, untreated control group (*n* = 6 for sham infected; *n* = 10 for infected); T, treated group (*n* = 20); s.e.m., standard error of the mean; Cov., covariate; Inf., infection; Ext., extract treatment; PT, post-treatment period (days 19–28); n.a, not applicable.

During the post-treatment period, there was a line × infection interaction, where infection resulted in a reduction in FI in slow-responder mice only (*P* < 0.05; Table 3). There was no effect of the extract on FI (*P* > 0.1). In a similar way to BW, there was a 3-way factorial line × infection × extract interaction on the FI–BW ratio (*P* < 0.05). The administration of the extract reduced the FI–BW ratio in sham-infected, fast-responder mice and increased the ratio in sham-infected, slow-responder mice (Table 3). Furthermore, the bark extract administration gave an increased FI–BW ratio in infected, fast-responder mice and a reduced FI–BW ratio in infected, slow-responder mice.

Spleen weight and SI weight were greater in the fast-responder line compared to the slow-responder line (*P* < 0.05, Table 3). In addition, these were all greater in infected animals compared to the sham-infected ones (*P* < 0.05). No effect of bark extract treatment or any interaction between line, infection and bark extract treatment on SI–CW ratio could be observed. There was a tendency of a significant line × infection × extract interaction on SI weight (*P* = 0.077). The pattern observed was similar to that observed for BW in the post-treatment period: the administration of bark extract resulted in a reduction in SI weight in infected, fast-responder mice and in the sham-infected, slow-responder mice but an increase in the SI weight of the sham-infected, fast-responder mice.

### Bark extract administration reduced the tolerance of infected, fast-responder mice but had no negative impact on the tolerance of the slow-responder mice

A significant negative correlation between TWC and CW (*r* = −0.5, *P* = 0.03), FEC and CW (*r* = −0.48, *P* = 0.03) and between EIC and CW (*r* = −0.55, *P* = 0.01) was evident in the fast-responder line that were administered the bark extract only (Table 4). Thus, the bark extract administration was responsible for the reduction in tolerance observed in the fast-responder mice.

**Table 4.** Pearson's correlations between total worm counts (TWC), fecal egg count (FEC) (days 22 and 28 post-infection), total eggs in colon (EIC) and carcase weight (CW) for the 2 selected mouse lines, infected with *H. bakeri* larvae untreated or treated with bark extract.

Correlation				TWC × CW		FEC × CW		EIC × CW	
Line	Treatment	*n*	DF	*r*	*P*	*r*	*P*	*r*	*P*
Fast responder	C	10	8	−0.42	0.230	−0.15	0.670	−0.29	0.420
	T	20	18	**−0.50**	**0.030**	**−0.48**	**0.030**	**−0.55**	**0.010**
Slow responder	C	10	8	0.00	0.980	−0.39	0.260	−0.28	0.440
	T	20	18	−0.16	0.510	−0.18	0.440	−0.24	0.299

C, untreated, infected control group; T, treated, infected group; *n*, number of animals in the calculation; DF, degree of freedom.

Significant correlations (*P* < 0.05) are demonstrated in bold.

There was no significant correlation between TWC, FEC or EIC, and CW in the slow-responder line (*r* = −0.24 to −0.16, *P* > 0.1), irrespective of bark treatment.

## Discussion

The administration of bark extract had a positive impact on the performance of infected, slow-responder mice as shown by a greater BW, CW and a lower FI–BW ratio in the post-treatment period. Bark extract administration however did not result in any benefits on the resistance of parasitized mice as measured by FEC, EIC, TWC, female worm fecundity and TWC–spleen weight relationship. Furthermore, bark administration had a negative effect on the tolerance of infected, fast-responder mice, whereas it had no impact on the tolerance of slow-responder mice.

To the best of our knowledge, this is the first study where the antiparasitic activity of bark extract from *P. sylvestris* was assessed against GINs *in vivo*. Under the conditions reported here, there was no impact of bark administration on the parasite load of mice. Previous studies on the impact of plant extracts on the resistance of *H. bakeri*-infected mice have reported variable results. For example, some studies have shown a reduction in FEC and/or TWC when treating *H. bakeri*-infected mice with various plant extracts at a level of 250–500 mg DM kg^−1^ BW (Enejoh *et al.*, 2015; Gutu, 2017), when others have shown no antiparasitic effect when drenching mice with plant extracts at the level of 125–500 mg DM kg^−1^ BW (Githiori *et al.*, 2003*a*, 2003*b*). In a study where *Nippostrongylus*-infected rats were treated with CT-rich quebracho extract in the feed (40 g DM extract kg^−1^ feed, equivalent to 6.8 g DM extract kg^−1^ BW), a reduction in the intestinal adult nematode population was observed compared to untreated controls (Butter *et al.*, 2001). In a previous study, we observed antiparasitic activity (expressed as reduced oocyst count) of water extracted spruce bark when drenching *Eimeria*-infected lambs with a CT dose 10 times higher than that achieved in the present study (Blomstrand *et al.*, 2022). In the current study, the achieved CT concentration administered to each animal was 4.2 and 2.0 mg CT mL^−1^ for each level, which was equivalent to approximately 0.14% of their daily FI. This is considerably lower compared to the amount of CT reported by Athanasiadou *et al.* (2001*a*) where sheep were drenched at approximately 6% of the daily FI. Based on this evidence, it seems plausible that the lack of an antiparasitic effect following bark extract administration in the current study may be attributed to the low CT concentration in the extracts administrated to mice. It could also be attributed to the types of CT available in pine; different types of CT are available in different plants. For example, quebracho extract, which has been previously shown to have strong anthelmintic activity against ovine nematodes *in vivo*, contains mainly profisetidins (Zhen *et al.*, 2021), whereas catechin/epicatechin was the dominating monomer of the CT in the pine bark extracts used in this study (Chylinski *et al.*, 2023).

It is not always possible to make comparisons of the activity of plant extracts across studies, as often the active compounds in the extracts are not known. For example, Githiori *et al.* (2003*a*), Enejoh *et al.* (2015) and Gutu (2017) used plant extracts of various origins at the levels of DM extract that appeared similar to those used in this study. Following administration with plant extracts, Githiori *et al.* (2003*a*) did not show biologically meaningful impact on fecal egg output in *Heligmosomoides polygyrus*-infected mice, whereas Enejoh *et al.* (2015) and Gutu (2017) demonstrated a reduction in FEC and TWC by approximately 70% following the extract administration in infected mice. None of these studies however associated the antiparasitic activity (or the lack of it) with particular compounds. This is one of the limitations when testing and reporting the anthelmintic activity of plant extracts, as different plants vary in their PSMs composition, many of which may have antiparasitic properties. Indeed, in a previous study we demonstrated a strong association between CT and other compounds with *in vitro* anthelmintic activity against *Teladorsagia circumcincta* and *T. colubriformis* (Chylinski *et al.*, 2023). It is critical that future studies investigating and reporting the anthelmintic activity of plant extracts also measure specific PSMs to facilitate across studies comparison and the progress of the field.

In addition to the relatively low level of CT, the lack of an anthelmintic effect may also be related to factors affecting the bioavailability of the active compounds in the different compartments of the gastrointestinal tract. Previous evidence has shown that anthelmintic efficacy attributed to CT was lower in sheep fed *ad libitum* compared to sheep fed restrictedly at 80% of their *ad libitum* intake (Athanasiadou *et al.*, 2001*a*). It was hypothesized that CT may need to reach a specific threshold in the gastrointestinal tract to be active and with *ad libitum* intake, which can lead to increased flow rates of digesta and reduced retention time of CT in the gastrointestinal tract, this may be difficult to achieve. As mice were fed *ad libitum* in the current study, the possibility that the retention time of the active compounds was reduced and thus were not accumulated at a level necessary to demonstrate an effect, cannot be disregarded. In addition, we have previously reported parasite species sensitivity to different PSMs (Athanasiadou *et al.*, 2021; Chylinski *et al.*, 2023), and a possible lack of *H. bakeri* sensitivity to the extract may also have had an impact on the lack of an antiparasitic activity observed in the present study.

Although the level of the active compounds in the extract may have been too low for any antiparasitic activity to be observed, it had a clear impact on the performance and the tolerance of the mice; bark extract administration had a positive impact on the performance of the infected, slow-responder animals as indicated by a higher BW and CW compared to the untreated control. This outcome is in support of the hypothesis; it is also consistent with the results of some of our previous studies (Athanasiadou *et al.*, 2001*a*), which showed that weaned infected lambs offered a diet supplemented with a CT-rich bark extract at 6% of the diet had a greater bodyweight gain and FI compared to the non-supplemented control animals. Athanasiadou *et al.* (2001*a*) suggested that increased FI may be a mechanism to compensate for a CT-generated loss of endogenous proteins. In the present study, the higher BW and CW were not accompanied by any change in FI in the bark-treated, slow-responder mice; it appears that these animals utilized the feed better. This observed effect might have been mediated *via* the intestinal microbiome. The intestinal microbiome is known to differ between these mouse lines, and it could have been affected differently by compounds in the bark (Turnbaugh *et al.*, 2006; Zhao *et al.*, 2019; Somayajulu *et al.*, 2021).

In the current study, only the infected, slow-responder mice, i.e. the most susceptible of all hosts to the negative consequences of parasitism, appeared to have benefited from the bark extract administration. Indeed, there was no impact on the performance of infected, fast-responder mice following bark extract administration; in addition, the performance of non-infected control animals was reduced following the bark extract administration. The latter is not an uncommon observation; CT-rich extract consumption has been associated with reduced FI and growth in non-parasitized rodent and sheep animals (Joslyn and Glick, 1969; Barry and McNabb, 1999; Athanasiadou *et al.*, 2001*a*, 2001*b*; Blomstrand *et al.*, 2022). When a CT-rich bark extract was offered at 8% of hosts’ FI, this resulted in lower FI and BW (Athanasiadou *et al.*, 2003). A significant reduction in FI and BW in parasitized lambs treated with pine bark extracts at a level equivalent to a CT content 6 times higher compared to that in the present mouse study has also been reported (Blomstrand *et al.*, 2022).

Athanasiadou and Kyriazakis (2004) have previously hypothesized that, despite these potentially negative consequences on their performance, parasitized animals can observe a beneficial impact from CT consumption if the positive (antiparasitic) effects of CT consumption outweigh the negative (anti-nutritional) effects. In this study, although there was no measurable antiparasitic activity observed, the most susceptible animals were still able to experience a positive impact of CT on their performance. It thus seems plausible that in the absence of a clear antiparasitic effect, a benefit on the performance of parasitized animals may be evident, but this may be dependent on hosts’ susceptibility to pathogens. The evolutionary benefit of this is evident, but further investigation into this hypothesis is required.

In addition to the positive impact of the bark extract administration on the performance of the infected, slow-responder mice, a significant negative impact on the tolerance of the infected, fast-responder mice was also observed; in these mice, tolerance, i.e. their ability to withstand the impact of parasitism, was reduced following the administration of bark. The 2 mice strains have been previously shown to vary in their tolerance to intestinal pathogens, with the fast responders showing higher tolerance to intestinal pathogens; the fast-responder strain appeared to be suffering the least from the impact of *H. bakeri* infection (Athanasiadou *et al.*, 2015). In another example, although both strains showed similar susceptibility to Shiga toxin and had similar bacteria counts in the intestine, the slow-responder mice (C57BL/6) experienced a greater impact on their fitness compared to the fast-responder mice (BALB/c) (Bernal *et al.*, 2021). Such differences in tolerance are thought to be associated with immune and inflammatory regulation (Medzhitov *et al.*, 2012). For example, BALB/c mice have been shown to have higher abundance and diversity of immunoglobulin A, increased microbiota diversity, and more efficient mucosal immune system compared to C57BL/6 mice as a consequence of better retinoic acid-mediated signalling (Goverse *et al.*, 2014; Fransen *et al.*, 2015). Compounds present in the bark extracts may interfere with such regulatory mechanisms and thus affect tolerance-regulated mechanisms to a different degree in the 2 strains; this indicates that fast-responder mice may be losing their advantage in tolerating parasite impact when drenched with bark. The reasons for this require further investigation.

In this study, the hypothesis was that infected, slow-responder mice (C57BL/6) would benefit more from the bark extract administration compared to mice of the fast-responder line (BALB/c). The predictions were that infected, bark extract-treated, slow-responder mice would experience a greater reduction in the parasitic burden, better performance and improved tolerance to the parasitic infection compared to the fast-responder mice. It was demonstrated that 2 out of 3 predictions supported the hypothesis. The infected, slow-responder mice experienced a benefit from the bark extract treatment compared to the fast-responder mice, with regards to performance and tolerance. There was no impact of bark administration on the level of parasitism in any animals, likely attributed to a low level of active compounds in the extracts. As 2 out of the 3 predictions in the hypothesis were met, the hypothesis is rejected.

Antiparasitic treatment may have various targets, for example to reduce the parasitic burden in the animals or the shedding of infective stages, hence reducing the infection pressure in the environment, or to improve the host animals’ tolerance and performance to the parasitism, helping the animals to withstand the impact of parasites (Athanasiadou *et al.*, 2007; Houdijk *et al.*, 2012). In mixed groups, where animals show variation in susceptibility and resistance, it may be important to reduce the infection pressure. However, a high infection pressure might be acceptable if all hosts have a high tolerance to the infection. The results showed that mice resistant to the parasitic infection may not benefit from treatment with the pine bark extract, but less resistant mice benefit more from such a treatment, through an improvement in their performance. If this activity is confirmed in livestock, it could have implications in parasite control strategies, for example it would be seen as an approach that targets genetically and physiologically susceptible animals and avoids treatment of more resistant animals.

## Data Availability

The datasets used and analysed during the current study are available from the corresponding author on reasonable request.
